# Editorial: Insights in male urology: 2021, volume 1

**DOI:** 10.3389/fruro.2023.1192753

**Published:** 2023-05-03

**Authors:** Giorgio Ivan Russo, Andrea Cocci, Ioannis Sokolakis, Arturo Lo Giudice, Maria Giovanna Asmundo

**Affiliations:** ^1^ Urology Section, Department of Surgery, University of Catania, Catania, Italy; ^2^ Department of Urology, University of Florence, Florence, Italy; ^3^ Department of Urology, Martha-Maria Hospital Nuremberg, Nuremberg, Germany

**Keywords:** erectile dysfunction, Peyronie disease, testicular chronic pain, testicular disease, urological health

This editorial of the Research Topic “*Insights in Male Urology: 2021*” encompasses a broad range of conditions affecting the male genitourinary system and aims to portray the last discoveries, new challenges and give an insight in the field of male urology.

Erectile dysfunction (ED) is defined as the persistent inability to obtain and maintain an erection that enables satisfactory sexual intercourse (1). ED is a very diffused condition affecting 6% to 64% of patients with a major prevalence among men of 40-79 years old (2). Although it is a non-lethal disorder, its psychological importance among couples and self-well-being makes interest in ED pathophysiology and treatment constantly improving.

Moreover, in recent years and increasing interest among the role of neuronal growth factor (NGF) in the pathogenesis of ED, as well as its potential therapeutic benefit (3, 4). According to studies, NGF may also play a pivotal role in the regulation of erection by its involvement in pathway that controls the erectile process (5). Evidences from animal models demonstrated that NGF promotes regeneration of damaged nerves of penis, especially in animals affected by diabetes, obesity and metabolic syndrome (Mets) (3).

The pilot case-control study conducted by Stabile et al. observed that patients with ED and contextual MetS has a reduction of NGF plasma concentration as well as a reduced tyrosine kinase A receptor (TrKA) expression. In the examined cohort of 8 patients affected by ED and MetS and 8 healthy patients, subjects were subdued to three blood drawing: the first one from the cubital vein; the second one from the corpora cavernosa after the intracavernous injection of 20 mg of Alprostadil; the third one was repeated from the cubital vein. Analysis did not registered NGF or TrKA augmentation after Alprostadil injection; however, lower NGF level were confirmed in case-group compared to control-group. Similarly, lower level of TrKA were registered as well.

Another condition affecting male urological health is Peyronie’s disease (PD) (6). PD is a condition in which scar tissue forms within the penis, causing it to bend or curve during erections; moreover, this condition may clearly lead to difficulties with sexual function and have substantial psychological implications. Moreover, pathophysiology and treatment options are not yet well defined, and they are under constant investigation (7).

In recent years, a growing interest among conservative treatment for PD has arisen; however, up to this moment, the only treatment approved by Food and Drug Administration (FDA) is collagenase Clostridium histolyticum (CCH). Several studies investigated the opportunity to apply alternative drug in this pathology treatment, such as hyaluronic acid (HA). Cocci et al. focused in demonstrating if a clinical efficacy of HA exists after 2 years of follow up. Authors performed a multicentric non-randomized study enrolling 244 patients affected by PD; subjects were divided into two groups and received respectively hyaluronic acid (HA) or verapamil extracavernous injection. Results showed not significantly difference among the two groups at follow up at 3-month, 1 year and 2 years in plaque size, penile curvature and IIEF-5; however, adjusted p-value for visual analogue scale (VAS) between groups was <0.01 at 3 months and not significant at 1 years and 2 years. Authors concluded that HA group was only slightly superior in improving penile curvature and in bothering of the symptoms.

Testicular diseases are also an important field in male urological panorama. In particular, chronic testicular pain is a condition characterized by persistent discomfort or pain in the scrotum region lasting for at least three months; outside the testicles, men affected by CTP are reported to have an high incidence of associated bothersome symptoms included urinary frequency (54%) and sexual dysfunction (55%) (8). As an obvious consequence, CPT may significantly impact on men quality of life because of the physical discomfort and emotional distress it generates (9). In the retrospective study conducted by Gandhi et al. investigating 2551 kidney donors, was observed that 2.12% of patients developed testicular pain 19 days after surgery. Moreover, authors observed that higher percentage of pain were registered among patients who underwent laparoscopic instead of open nephrectomy (3.6% vs 1.1%). According to authors, this difference may be explained by the attitude of performing gonadal vein ligation during laparoscopy; this procedure may lead to associated lymphatic channel ligation and testicular congestion.

On the other side, testicular damage may be caused by a direct and well recognized event that could as well, lead to physical and emotional damage. Moreover, a wide range of sexual dysfunction and long-lasting effects that could result in infertility are described in patients affected by traumatic testicular injuries (TTI) (10). For these reasons, prompt evaluation and appropriate treatments are fundamental. TTI often result in morbidity associated with genital injury or, in worst cases, in organ loss. In the retrospective study conducted by Mitchell et al., a comparison between patients from a rural area presenting with TTI and TTI from the National Trauma Data Bank (NTDB^®^) was performed. Data showed that blunt trauma has a higher incidence among rural area patients compared to NTDB (91.3% vs 50.5%); moreover, the incidence of scrotal exploration rate and subsequent orchiectomy is higher among residents from the rural area (90.4% vs 48.3% and 52.4% vs 23.4% respectively). These findings may be explained by the difficult access to hospital, the lack of rapid transfer time and successive care by subspecialist.

Therefore, the editors decided to give space, in their special issue, to papers exploring male urology in 2021 with a special focus on a range of conditions affecting genitourinary system and men well-being including ED, PD and testicular diseases with a particular focus on testicular chronic pain.

Since the conditions analyzed are as common as sensitive, increasing importance had been dedicated to minimally invasive surgical approaches as well as to innovative medical treatment. As an instance, in the broad field of male infertility, that may also be caused by chronic or acute testicle damage, the employment of latest technology as 4K 3D microscope or newest testing tools such as epigenetic sperm examination could maximize the chance to succeed in ART (assistive reproductive technology) and, indirectly, on male general well-being.

However, lacking consensus on newest treatment and surgical technique, makes further research essential to refine and define the best management and approach to the listed conditions.

## Author contributions

Writing—original draft preparation, AG, MA, and GR; writing—review and editing, MA and GR; supervision, IS, AC, and GR. All authors have read and agreed to the published version of the manuscript.
